# Treatment for Brodie’s abscess of the radius in an adolescent: A case report

**DOI:** 10.1016/j.ijscr.2020.06.106

**Published:** 2020-07-10

**Authors:** Takahiro Ushijima, Ken Arai

**Affiliations:** Department of Orthopaedic Surgery, Iizuka Hospital, 3-83, Yoshio Town, Iizuka City, Fukuoka Prefecture, 820-8505, Japan

**Keywords:** Brodie’s abscess, Chronic osteomyelitis, Curettage

## Abstract

**Introduction:**

Brodie’s abscess is an uncommon type subacute osteomyelitis. It is typically localized in the metaphysis of tubular bones, particularly in the lower extremities. We herein report a rare case of the abscess appearing in the upper extremities. Furthermore, we successfully treated the large abscess without autogenous bone grafting.

**Presentation of case:**

14-year-old female presented with pain and swelling on the right forearm. Plain radiograph and CT scan indicated a 10 cm longitudinal cortical bone hypertrophy and a well-defined radiolucent lesion in the diaphysis of the right radius. MRI demonstrated that the lesion was hypointense on T1-weighted imaging and hyperintense on T2-weighted imaging inside as well as outside the bone marrow of the radius. Laboratory data showed no inflammatory response, but *Staphylococcus aureu*s was detected by biopsy. We diagnosed Brodie’s abscess of the radius, and performed definitive surgery. Infected bone marrow was curetted and a bony sequestrum inside the cortical bone was harvested. We did not use autogenous bone grafting, since the upper extremities are areas of unloaded bone. Postoperative administration of antibiotics was subsequently performed. One year after surgery, the patient was asymptomatic and there were no complications or signs of infection recurrence.

**Conclusion:**

We diagnosed and surgically treated a rare case of Brodie’s abscess of the radius in an adolescent. An abscess with large cavity is usually treated by curettage and autogenous cancellous bone grafting. However, since the upper extremities are areas of unloaded bone, we successfully treated the abscess by debridement without bone grafting.

## Highlights

• Brodie’s abscess occurring in upper extremities is rare.
• Penumbra sign identified on MRI is a useful finding for diagnosis.
• We recommend that bone grafting after curettage of the abscess cavity is not always necessary for upper extremities.

## 1 Introduction

Brodie’s abscess was reported as an uncommon type subacute osteomyelitis demonstrated by bone cavity surrounded by bone sclerosis [1,2]. The abscess is typically localized in the metaphysis of tubular bones, particularly in the lower extremities [3,4]. Few studies have reported Brodie’s abscess in the upper extremities. It is often difficult to diagnose the abscess because there are no characteristic findings on a plain radiograph and no obvious inflammatory response. An abscess with large cavity is usually treated by curettage and autogenous cancellous bone grafting [3]. Here, we diagnosed a rare case of Brodie’s abscess appearing in the upper extremities and successfully treated it without bone grafting. This case has been reported in line with SCARE guidelines [5].

## 2 Presentation of case

**Fig. 1:**
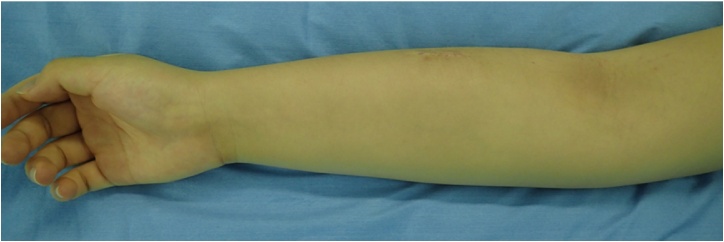
Appearance of right forearm. Right forearm is swollen, but there is no erythema or flare. A biopsy scar is observable in the middle of the external ventral portion of the forearm.

**Fig. 2:**
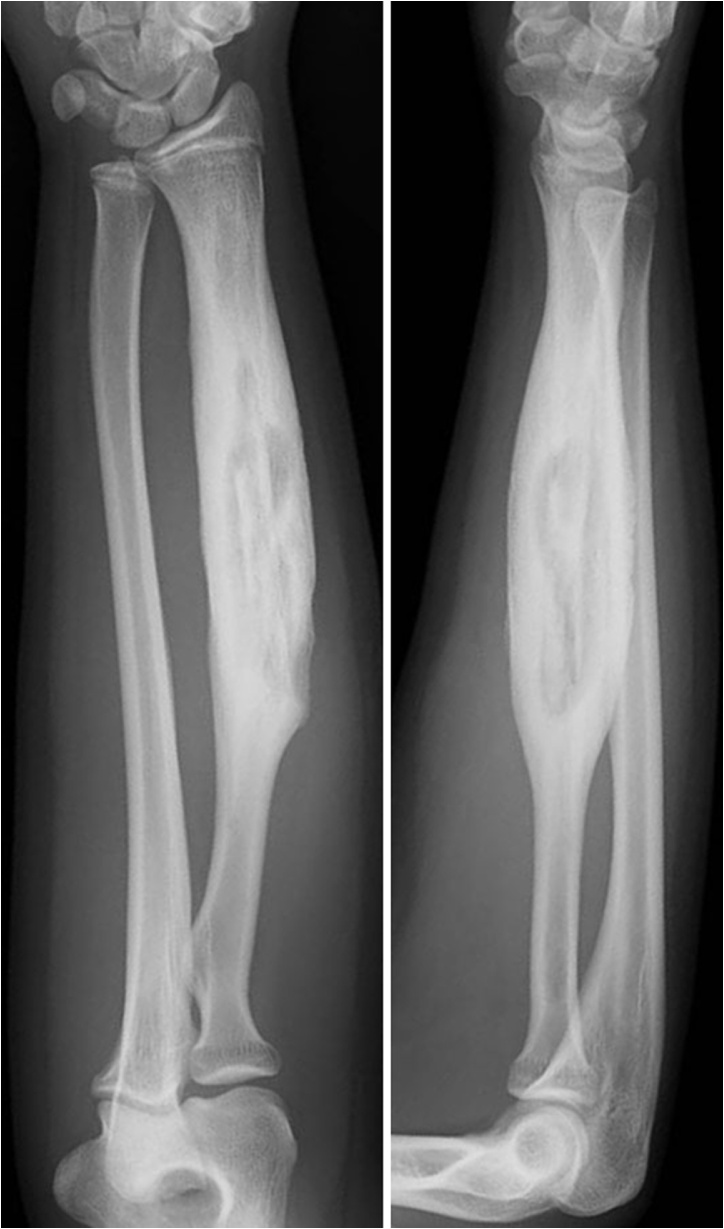
Plain radiograph. A plain radiograph shows hypertrophic cortical bone and a radiolucent lesion in the diaphysis of the right radius.

**Fig. 3:**
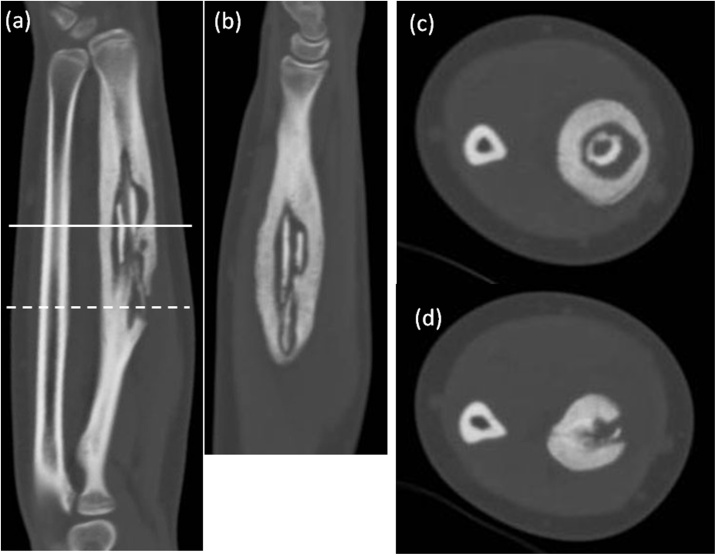
Plain CT. A CT scan shows a longitudinal translucent lesion, a cortical defect of the middle diaphysis, and cortical bone in the bone marrow of the radius. The solid and dotted lines in figure (a) correspond to figure (c) and figure (d), respectively.

**Fig. 4:**
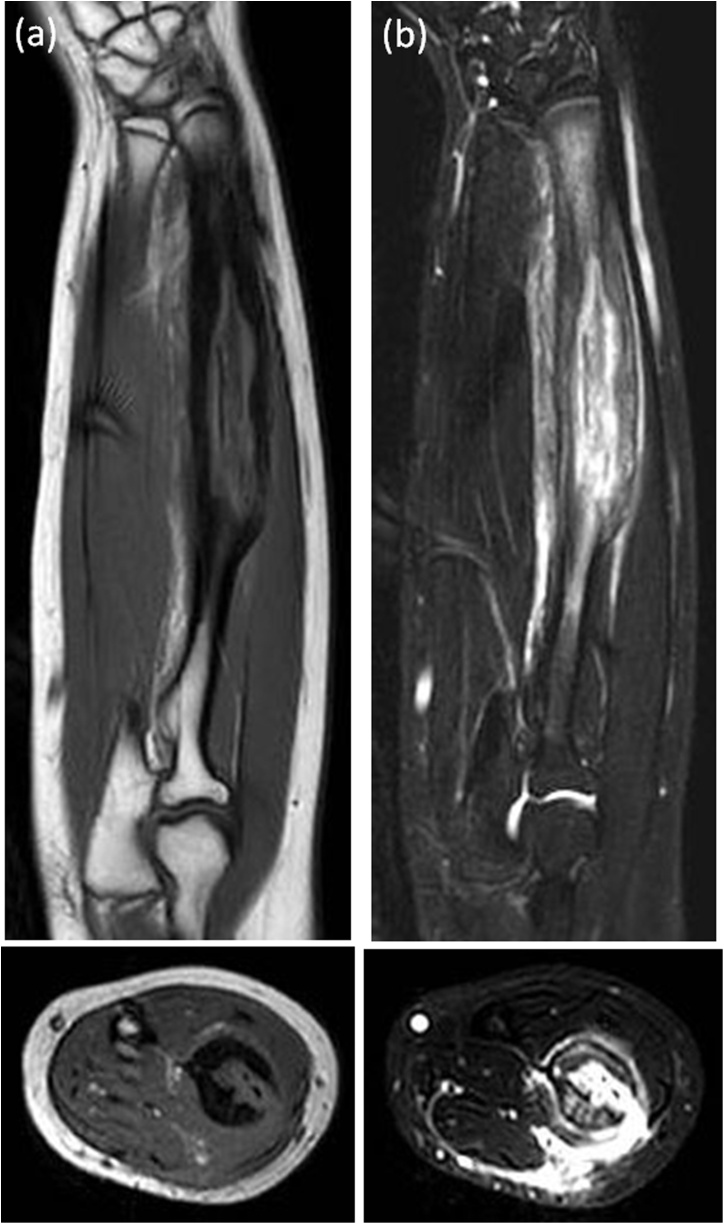
MRI. (a), (b) An MRI shows that the lesion is hypointense on T1-weighted imaging and hyperintense on T2-weighted imaging inside the radius. (c), (d) Extraosseous abscess is well visualized as a hypointense lesion on hyperintense lesion on T2WI outside the radius.

A 14-year-old female presented with pain and swelling on the volar aspect of her right distal forearm 2 months previously and was referred to our tertiary care center for further evaluation. The patient had not suffered trauma before the symptoms appeared and did not have any past medical or surgical history. Upon physical examination, the circumference of the right forearm was 15 mm larger than that of the left one, but there was no erythema or flare (Fig. 1). Obvious restriction of range of motion and neurovascular deficit in the hand was not observed. A plain radiograph indicated cortical bone hypertrophy and a well-defined radiolucent lesion in the diaphysis of the right radius (Fig. 2). A CT scan showed a 10 cm longitudinal translucent lesion, a cortical defect on the volar side of the middle diaphysis, and cortical bone in the bone marrow of the radius (Fig. 3). MRI demonstrated that the lesion was hypointense on T1-weighted imaging (T1WI) and hyperintense on T2-weighted imaging (T2WI) inside the bone marrow of the radius (Fig. 4). A hyperintense lesion on T2WI was also found outside the radius, indicating an extraosseous abscess. The penumbra sign [6], indicated by the lining of an abscess cavity showing hyperintensity relative to the main abscess contents on T1WI, was also identified.

Laboratory investigations showed a white blood cell (WBC) count of 6060/μl, a C-reactive protein (CRP) level of 0.03 mg/dl, and a normal level of sedimentation rate (9 mm/1 h). The patient did not have a fever.

Biopsy confirmed that *Staphylococcus aureus* was detected from the extraosseous abscess. Sensitivity analysis revealed the bacteria was sensitive to all antibiotics. No obvious malignant cells were found.

According to these findings, we diagnosed Brodie’s abscess of the radius. This case was considered chronic osteomyelitis, therefore, surgical treatment was undertaken.

**Fig. 5:**
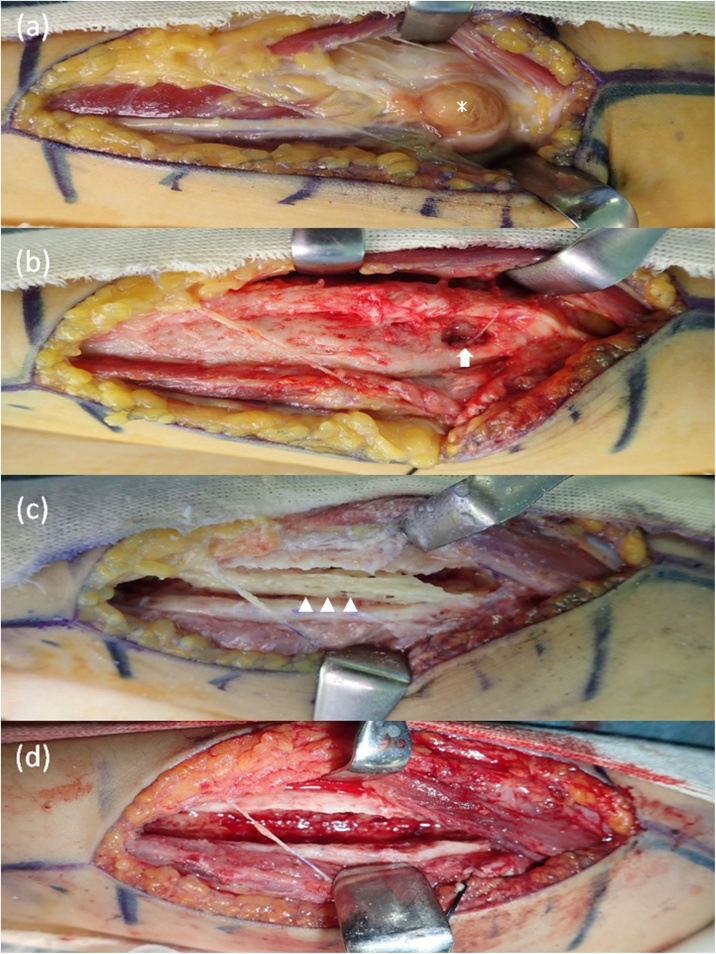
Intraoperative findings. (a) An extraosseous abscess exists beside the pronator teres (＊). (b) A cortical bone defect at the back of the abscess is found (arrow). The abscess, enclosed by a capsule, contained yellowish-brown soft tissue. (c) There is a bony sequestrum in the diaphysis of the radius (arrow head). (d) Debridement was performed in the canal of the radius, and good bleeding from the bone was also found.

Like the Henry approach, a longitudinal skin incision along the flexor carpi radialis was made on the volar aspect of the forearm. An extraosseous abscess was found beside the pronator teres (Fig. 5a), and there was a 5 mm cortical bone defect at the back of the abscess (Fig. 5b). The abscess, enclosed by a capsule, contained yellowish-brown soft tissue. The volar aspect of the cortex of the radius was curetted and enlarged to 10 mm × 90 mm. A bony sequestrum was found in the enlarged diaphysis of the radius (Fig. 5c). Debridement was undertaken in the canal of the radius (Fig. 5d). Previous reports indicated that bone grafting after curettage is necessary for large abscesses [3,7]. However, since the upper extremities are areas of unloaded bone, autogenous bone grafting was not performed. *Staphylococcus aureus* was also found in the intraoperative culture. The right upper arm was fixed by long arm cast for 2 weeks after surgery and the patient was subsequently forbidden from strenuous exercise for 6 months.

**Fig. 6:**
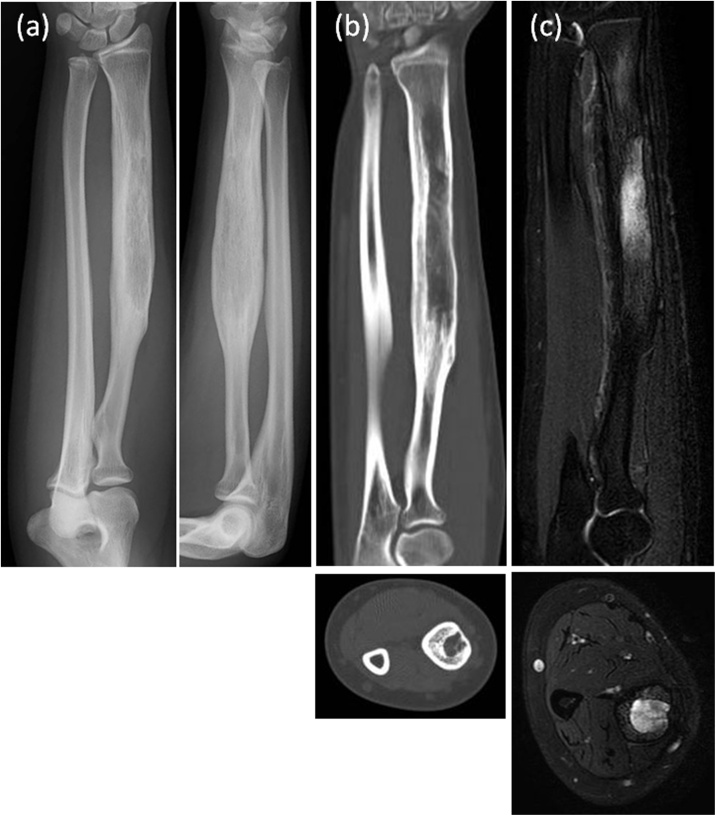
Image findings one year after surgery. (a), (b) Plain radiograph and CT scan show the disappearance of the sclerotic lesion and the recovery of the trabecular structure of the radius. (c) An MRI shows that the extraosseous abscess has disappeared.

After consultation with an infectious control team, cefazolin 6 g per day for 2 weeks, followed by clindamycin 1.8 g per a day for 6 weeks were administrated. Laboratory examinations indicated that transient inflammatory response was increased postoperatively, but quickly fell into the normal range. Afterward, we confirmed no signs of infection recurrence by laboratory and radiographic examination once a month. One year after surgery, the patient had no pain or swelling, and achieved full range of motion of her wrist and elbow. Deformity and impaired bone growth of the upper extremities did not occur. Plain radiograph and CT scan showed the disappearance of the sclerotic lesion and the recovery of the trabecular structure of the radius (Fig. 6a, b). Moreover, an MRI indicated that the extraosseous abscess had disappeared (Fig. 6b).

## 3 Discussion

Brodie’s abscess was first reported as subacute osteomyelitis in the tibia without any acute symptoms [1]. Laboratory investigations for detecting inflammatory response were usually within the normal limits [8]. Brodie’s abscess appears more commonly in the lower extremities, especially in the metaphysis of the tibia or femur. Abscesses occurring in the upper extremities are rare. Olasinde et al. [9] reported that there was only one case of Brodie’s abscess of the radius among a series of 20 cases. Takeuchi et al. [7] also reported one case of Brodie’s abscess occurring in the radius.

It is difficult to diagnose osteomyelitis by imaging investigations. A plain radiograph is less helpful than MRI in distinguishing it from another disease, such as a bone tumor. Grey et al. [6] reported that the “penumbra sign” of Brodie’s abscess was the characteristic finding on MRI. The “penumbra sign” is identified on T1WI as a discrete peripheral zone of marginally higher signal intensity than the abscess cavity and surrounding marrow sclerosis, and lower signal intensity than the fatty bone marrow. On the other hand, the “double line sign” that consists of two lines located at the periphery of the cavity, an outer low signal line and inner high signal line, has been reported as a T2WI feature [10]. In our case, the “penumbra sign” on T1WI was also detected.

Regarding management of Brodie’s abscess, several treatments have been reported. It has been indicated that the medical costs, complications and length of hospital stay for surgical debridement and postoperative antibiotics are lower than those of conservative treatment by antibiotics only [11]. In our case, we performed operative treatment since osteomyelitis was chronic and antibiotics might not be effective on bony sequestrum inside the cortical bone. The use of antibiotic-impregnated beads is one of therapeutic approaches for Brodie’s abscess [12]. However, we considered removal of the bony sequestrum and radical curettage was most important for the operative procedure. Stephens and MacAuley [3] indicated that curettage of the abscess cavity with cancellous bone grafting has been reserved mainly for those with large cavity diameters of greater than 30 mm. However, these surgical treatments are toward Brodie’s abscesses of the lower extremities. We are of the opinion that autogenous bone grafting after curettage is not necessary for unloaded bone.

## 4 Conclusion

We experienced a case of Brodie’s abscess of the radius in an adolescent, and surgically treated it by debridement of the abscess without autogenous cancellous bone grafting.

## Declaration of Competing Interest

We have no conflicts to declare on this literature.

## Sources of funding

There are no funding sources.

## Ethical approval

Case reports are exempted from ethical approval according to our institution guidelines.

## Consent

Written informed consent was obtained from the patient for publication of this case report and accompanying images. A copy of the written consent is available for review by the Editor-in-Chief of this journal on request.

## Author contribution

Takahiro Ushijima, Ken Arai: Surgeons.

Takahiro Ushijima, Ken Arai: Conception of reporting case, data recording, and drafting.

Takahiro Ushijima: Writing the paper.

## Registration of research studies

This case report is not applicable.

## Guarantor

Takahiro Ushijima.

## Provenance and peer review

Not commissioned, externally peer-reviewed.
